# Can we change binge eating behaviour by interventions addressing food-related impulsivity? A systematic review

**DOI:** 10.1186/s40337-021-00384-x

**Published:** 2021-03-18

**Authors:** Başak İnce, Johanna Schlatter, Sebastian Max, Christian Plewnia, Stephan Zipfel, Katrin Elisabeth Giel, Kathrin Schag

**Affiliations:** 1 Department of Psychology, Haliç University, Istanbul, Turkey; 2 Department of Psychosomatic Medicine and Psychotherapy, University Hospital Tübingen, Osianderstraße 5, 72076 Tübingen, Germany; 3 Department of Psychiatry and Psychotherapy, Neurophysiology & Interventional Neuropsychiatry, University of Tübingen, Tübingen, Germany; 4 Competence Center of Eating Disorders Tübingen (KOMET), Tübingen, Germany

**Keywords:** Binge eating, Eating behaviour, Food, Impulsivity, Treatment, Training, Psychotherapy, Pharmacotherapy, Neurostimulation

## Abstract

**Background:**

An extensive amount of research has underlined the potential role of impulsivity in the development and maintenance of binge eating behaviour. Food-related impulsivity has particularly received attention given its close relationship with overeating and binge eating episodes. Besides the available evidence, our understanding regarding the effectiveness of treatment modalities for binge eating targeting impulsivity and related constructs (e.g., food craving, inhibitory control, and reward sensitivity) is limited. Thus, this systematic review aimed to investigate whether binge eating behaviour is changeable by interventions that are impulsivity-focused and food-related and whether one of these interventions is superior to the others.

**Method:**

A search on PubMed and PsycINFO was performed for relevant articles published up to September 2020. Studies delivering food-related impulsivity treatment to individuals suffering from binge eating episodes and including a control condition without this treatment were investigated. Following the search, 15 studies meeting the eligibility criteria were analysed.

**Results:**

Analyses revealed that available impulsivity-focused approaches can be categorised as psychotherapy, pharmacotherapy, computer-assisted cognitive training, and direct neuromodulation interventions. Regarding their effectiveness, it appeared that all of these approaches might be promising to change food-related impulsivity in individuals with binge eating episodes, particularly to decrease binge eating symptoms. However, a superior intervention approach in this early state of evidence could not be determined, although food-related cue exposure, transcranial direct current stimulation, and the combination of several interventions seem fruitful.

**Conclusion:**

Efforts to treat binge eating behaviour with interventions focusing on food-related impulsivity appear to be promising, particularly concerning binge eating frequency, and also for food craving and inhibitory control. Given limited research and varying methods, it was not possible to conclude whether one impulsivity-focused intervention can be considered superior to others.

## Plain English summary

As one of the core symptoms of bulimia nervosa and binge eating disorder, binge eating behaviour negatively influences the physical and psychosocial well-being of a significant number of people. Current first-line treatments for bulimia nervosa and binge eating disorder are effective, but often do not result in sustainable remission for a substantial number of patients. Existing treatments targeting binge eating might benefit from interventions directly targeting impulsivity, particularly food-related impulsivity, which can be a potential etiological and/or maintaining factor for binge eating behaviour. With this systematic review investigating novel impulsivity-focused treatments for individuals with binge eating behaviour, promising approaches to change food-related impulsivity are described. The included studies use several impulsivity-focused treatments, ranging from specific psychotherapy that includes so-called cue exposure treatment, as well as computer training, pharmacotherapy, or direct stimulation of the brain with special equipment. Despite the limited number of studies and lack of data available to conclude the superiority of one of these approaches, the reviewed treatments seem promising to improve treatment outcomes.

## Background

Binge eating behaviour refers to the consumption of a large amount of food in a short period of time accompanied by feelings of loss of control over eating [1]. Even though binge eating behaviour can be observed in many individuals irrespective of an eating disorder (ED) diagnosis (e.g., people with obesity or emotional eating), it is considered to be the core feature of bulimia nervosa (BN) and binge eating disorder (BED). BN and BED are frequently presenting EDs affecting people worldwide. According to the data collected from 14 countries for the World Mental Health Survey of the World Health Organization, lifetime prevalence estimates were 1.0% for BN and 1.9% for BED [2]. Since binge eating behaviour causes weight gain, medical complications, and psychosocial impairments [3], it is important to explore and further understand the underlying and maintaining mechanisms behind this dysfunctional behaviour.

According to systematic reviews and meta-analyses, a great number of studies have identified the personality trait impulsivity as a potential aetiological and/or maintaining factor for binge eating behaviour [4–6]. Impulsivity is not only a personality trait, but also a significant construct for the understanding and diagnosis of a variety of psychological illnesses (e.g., attention-deficit/hyperactivity disorder, borderline personality disorder, and substance use disorder) [7]. Moeller and colleagues [8] defined impulsivity as “a predisposition toward rapid, unplanned reactions to internal or external stimuli without regard to the negative consequences of these reactions to the impulsive individual or others” (p. 1784). More recently, Gullo and colleagues [9] investigated the facets of impulsivity by considering different models and summarised that reward sensitivity and disinhibition are the aspects of impulsivity that play the main role in addictive-like behaviour. Negative urgency, i.e. the tendency to show impulsive behaviour particularly in negative mood is currently accepted as a factor of impulsivity, following reward sensitivity and disinhibition [9].

In particular, food-related impulsivity has received attention given its close relationship with binge eating behaviour [5]. Dawe and Loxton [10] initially proposed a relationship between food-related impulsive tendencies and binge eating. According to the authors, individuals who binge eat suffer from increased food craving, i.e. an intense desire to eat particular foods as they perceive food and related stimuli highly rewarding [11]. Having a high level of reward sensitivity towards food-related stimuli could increase the likelihood of binge eating and decrease the ability to inhibit or control eating [10]. Similarly, Treasure and colleagues [12] propose that individuals with binge eating behaviour have a hyper-responsive reward system and impaired inhibitory control towards food-related cues. Regardless of the type of instrument used, namely self-report measures, behavioural tasks, and electroencephalography (EEG), the evidence supports a positive association between increased impulsivity and binge eating behaviour in both healthy and clinical samples [5, 8]. For example, electrocortical process analyses in EEG studies have shown alterations regarding conflict processing, inhibitory control deficits, and higher levels of frontal beta activity which has been positively associated with disinhibited eating [13, 14]. Studies investigating the neurobiological basis of binge eating behaviour report an enhanced attentional bias towards food stimuli, alterations in the reward system, and impairments in cognitive functions like poor inhibitory control skills towards food [15, 16]. Moreover, evidence suggests that individuals with high levels of negative urgency are more likely to consider food as a way of coping with negative emotions, thus engaging in binge eating behaviour [17, 18]. Taken together, this biopsychological model of food-related impulsivity including increased reward sensitivity, disinhibition, and negative urgency strongly resembles the three-pathway model of alcohol craving [19] and hints that substance addictions and impulsive eating behaviours might share similar psychobiological processes.

Moreover, evidence suggests that impulsivity predicts the development of binge eating behaviour [20–22]. For example, negative urgency together with negative affect predicts binge eating onset in a longitudinal study examining school children over a time span of 1 year [22]. Impulsivity seems also to increase the risk for other mental disorders like substance use disorders and additional psychological problems like self-harming behaviours and negative affect [23]. Lastly, impulsivity also predicts treatment outcomes, with higher levels of impulsivity interfering with treatment success by making it difficult to implement newly acquired skills or resulting in possible relapse [24].

Cognitive-behavioural therapy (CBT), interpersonal therapy (IPT), and dialectical behaviour therapy (DBT) are considered first-line treatments for individuals with BN and BED [25–27]. However, meta-analyses and review studies indicate that available conventional treatments have difficulties in decreasing binge eating behaviour, with up to 50% of patients not benefiting from these treatments and remaining symptomatic [27, 28]. One reason for this might be that although theoretical models and research concerning binge eating behaviour emphasize impulsivity, the translation of this evidence into treatment is still limited. Impulsivity and related constructs are hardly targeted in treatment approaches for binge eating [29]. Thus, integrating impulsivity into treatment for binge eating behaviour could be a fruitful approach to improve treatment outcomes and decrease relapse rates.

In this regard, this study aims to systematically review existing impulsivity treatment approaches for individuals with binge eating behaviour, in order to discuss their effectiveness and provide recommendations for future research and clinical work. In particular, we investigated interventions which use food stimuli as they directly target impulsive eating behaviour, i.e. binge eating. Such impulsive eating behaviours might also be more easily modifiable and measurable than the underlying trait. We define treatment in this article as any form of intervention targeting binge eating behaviour, i.e. in forms of psychotherapeutic, computer-assisted cognitive training, neuromodulation or pharmacological approaches.

Taken together, we aim to answer the following research questions:

1. Is there any evidence that food-related impulsivity can be changed by impulsivity-focused interventions in individuals with binge eating behaviour?
2. If so, is there any evidence to conclude that one of these interventions is superior to the others?

## Materials and methods

This systematic review was conducted based on the PRISMA-Statement [30, 31].

### Search strategy

A search on the scientific databases PubMed and PsycINFO and additional hand search was performed for relevant articles published up to September 2020 with no starting date. For the PubMed search, the following search terms were used:

(binge-eating disorder [MeSH Terms] OR “binge eating”[Title/Abstract] OR „binge-eating“[Title/Abstract] OR “BED“[Title/Abstract] OR bulimia [MeSH Terms] OR bulimia [Title/Abstract] OR Hyperphagia [MeSH Terms] OR Hyperphagia [Title/Abstract] OR overeating [Title/Abstract] OR overeating [MeSh Terms]) AND (impulsive behavior [MeSH Terms] OR impulsiv*[Title/Abstract] OR impulsivity [MeSH Terms] OR reward [MeSH Terms] OR reward [Title/Abstract] OR disinhibit*[Title/Abstract] OR “loss of control“[Title/Abstract] OR “inhibition psychology“[MeSH Terms]) AND (therapy [MeSH Terms] OR therapy [Title/Abstract] OR “behavioural change “[MeSH Terms] OR behavioural change [Title/Abstract] OR Intervention [Title/Abstract] OR Intervention [MeSH Terms] OR “Stop Signal “[Title/Abstract] OR “Stop Signal “[MeSH Terms] OR Training [Title/Abstract] OR “training support”[MeSH Terms] OR behavioural modification [MeSH Terms] OR behavioural modification [Title/Abstract] OR “transcranial magnetic stimulation “[Title/Abstract] OR “transcranial direct current stimulation “[Title/Abstract] OR “vagus nerve stimulation “[Title/Abstract] OR “deep brain stimulation “[Title/Abstract]).

For the PsycINFO search, the following search terms were used with a filter for academic journals:

(TI (binge eating OR binge-eating OR BED OR Bulimia OR Hyperphagia OR overeating) OR AB (binge eating OR binge-eating OR BED OR Bulimia OR Hyperphagia OR overeating)) AND (TI (impulsiv* OR reward OR disinhibit* OR “loss of control”) OR AB (impulsive behavior OR impulsiv* OR reward OR disinhibit* OR “loss of control”)) AND (TI (therapy OR behavioural change OR behavioral change OR intervention OR training OR behavioural modification OR transcranial magnetic stimulation OR transcranial direct current stimulation OR vagus nerve stimulation OR deep brain stimulation) OR AB (therapy OR behavioural change OR behavioral change OR intervention OR training OR behavioural modification OR transcranial magnetic stimulation OR transcranial direct current stimulation OR vagus nerve stimulation OR deep brain stimulation)).

### Eligibility criteria

As recommended in the PRISMA statement, eligibility was based on the PICOS criteria: participants, interventions, comparators, outcome and study design [31].

#### Participants

Studies including individuals of any age or gender who suffer from binge eating episodes with a diagnosis of BED, BN, EDNOS, OSFED or subclinical binge eating behaviour. Studies were excluded if the subjects suffer from neurological disorders (e.g., Parkinson’s disease).

#### Interventions

In order to be included, studies must offer some form of impulsivity-focused intervention that targets food-related impulsivity (e.g., group therapy, neuromodulation, or computer-assisted cognitive training). The term “impulsivity-focused intervention” requires at least one factor of impulsivity to be targeted in the treatment, e.g. reward sensitivity, inhibitory control, and/or negative urgency.

#### Comparators

As a comparison group, studies with a control group in which participants did not receive a food-related impulsivity-focused treatment were included. Another intervention in the control group (e.g. treatment as usual) was possible, though it was not a necessary inclusion criterion. Studies with within-subject comparisons, i.e. where sessions with the food-related impulsivity-focused treatment were compared with sessions without this treatment in the same subjects, were also included.

#### Outcome

Studies were considered to be eligible if they included at least one measure related to food-related impulsivity. For example, this could be the assessment of binge eating episodes by standardised interviews or questionnaires (EDE, EDEQ), or by experimental paradigms, e.g. Stop Signal task or Go/No Go task with the presentation of food stimuli. Changes of trait impulsivity by interviews, self-reports or experimental paradigms were also reported though this was not a necessary inclusion criterion.

#### Study design

Clinical studies, experimental studies, and observational studies were included. Case studies were excluded.

### Study selection and data collection

Three authors of the present article independently assessed the eligibility of the articles that were identified following the database search based on the eligibility criteria. The first and the second author independently screened the articles by scanning their titles and abstracts and removed duplicated articles. These two authors and the last author performed the evaluation of the full texts of studies that were potentially relevant to the eligibility criteria. In the case of contrary opinions, the single studies were discussed with the last author.

Extracted data for the included articles are displayed in Table 1 and contain: (i) characteristics of the study participants (diagnosis, number of participants and type of control group); (ii) characteristics of the interventions (session numbers, format); (iii) type of impulsivity and related measures; and (iv) summary of main findings.

**Table 1:** Summary of the studies investigating impulsivity focused interventions to reduce binge eating behaviour

Study	Sample	Intervention	Dose	Impulsivity and Related Measures	Summary of Findings
***Psychotherapy approach***					
**Ferrer-García et al. (2017)** [3]	*N* = 64 adults with BED or BN after CBT fails	Virtual Reality & Cue Exposure Therapy (VR-CET) vs. Additional CBT (A-CBT)	6 individual sessions twice a week	1. EDE (binge eating episodes)	1. Both groups reduced binge eating, VR - CET was significantly superior compared to A-CBT regarding the reduction of binge eating frequency and achievement in abstinence from binge eating episodes (53% vs. 25%).
				2. EDE (purging episodes)	2. Both groups reduced purging behaviour, VR-CET was also significantly superior to A-CBT for achievement in abstinence from purging episodes (75% vs. 31.5%).
				3. EDI - Bulimia subscale (self-reported binge eating tendency)	3. Both groups improved self-reported binge eating tendency, but VR-CET was significantly superior to A-CBT.
				4. FCQ (state and trait version)	4. Both groups reduced both state and trait food craving, but VR-CET was significantly superior to A-CBT.
**Preuss et al. (2017)** [32]	*N* = 69 treatment seeking obesity patients (40.6% OSFED, 33.3% BED)	ImpulsE (psychotherapeutic treatment to increase inhibitory control and emotion regulation) + food-specific stop-signal inhibition training vs. TAU (CBT for obesity and BED)	10, 100-min sessions in group format	1. EDEQ (frequency of episodes of overeating and objective binge eating)	1. Frequency of disinhibited overeating decreased in both conditions. Significant binge eating reduction in patients with BED at post-treatment and 3-month FU in ImpulsE group, no change in TAU.
				2. SST with food stimuli (inhibitory control)	2. Significantly greater reduction in inhibitory control in ImpulsE group compared with TAU.
				3. UPPS Impulsive Behaviour Scale (urgency, lack of premeditation, lack of perseverance and sensation seeking)	3. Perceived lack of perseverance and urgency significantly decreased in both groups.
**Schag et al. (2019)** [33]	*N* = 80 adults with BED	IMPULS (impulsivity focused group intervention) vs. Control group without intervention	8, 90-min sessions in group format	1. EDE (binge eating episodes)	1. Binge eating episodes in the past 4 weeks were significantly reduced in both groups at the end of treatment. Binge eating was reduced more in the IMPULS group vs. control group at 3 months follow up.
				2. DEBQ (external eating subscale)	2. External eating was reduced more in IMPULS group at the end of treatment and follow up. Control group showed reduction only at follow up.
				3. BIS-15 & BIS/BAS (trait impulsivity)	3. Trait impulsivity was not significantly reduced in any group.
***Pharmacotherapy approach***					
**Chao et al. (2019)** [34]	*N* = 150 obese adults with binge eating	IBT-alone vs. IBT-liraglutide vs. Multicomponent (IBT + liraglutide + portion-controlled diet)	21 sessions of IBT vs. 21 sessions of IBT + 3.0 mg/d as a once-daily vs. 21 sessions of IBT + 3.0 mg/d as a once-daily + 12-week, 1000- to 1200-kcal/d diet	1. EDEQ (binge eating episodes)	1. At week 24, the IBT-liraglutide and multicomponent groups showed a significant within-group mean decline. The multicomponent group had a greater decrease compared to the IBT-alone group at week 24. All groups had significant within-group declines in binge eating at week 52, with a greater decline in the multicomponent group.
				2. FCI (frequency of food cravings)	2. All groups had significant and similar declines in total food cravings at both 24 and 52 weeks.
				3. EDI (dietary disinhibition)	3. All groups had a significant within-group decline at week 24 and 52. At week 24, the IBT-alone and IBT-liraglutide groups did not differ, but the decline was significantly less in the IBT-alone group compared to the multicomponent group. At week 52, there was no significant group difference.
**Da Porto et al. (2020)** [35]	*N =* 60 type 2 diabetic outpatients with BED	dulaglutide vs. gliclazide modified release +metformin	dulaglutide 150 mg/week vs. gliclazide modified release 60 mg/day + metformin (dosage 2–3 g/day)	1. BES (binge eating)	1. Binge eating behaviour was only significantly reduced in the dulaglutide group.
**Quilty et al. (2019)** [36]	*N* = 49 women with BED	Psychostimulant medication (Methylphenidate) vs. CBT TAU	12 weeks of medication usage (initial dosage: 18 mg; final dosage 72 mg) vs. 12 weekly individual sessions for CBT	1. EDE (objective binge eating)	1. Objective binge episodes decreased in both groups, with no treatment effect.
				2. BES (subjective binge eating)	2. Subjective binge episodes decreased in both conditions, with no treatment effect.
				3. The UPPS Impulsive Behaviour Scale (urgency, lack of premeditation, lack of perseverance and sensation seeking)	3. Perseverance and negative urgency scores decreased in both conditions over time with no treatment effect. Higher levels of UPPS perseverance and negative urgency scores were associated with a better treatment outcome in both conditions.
***Computer-assisted cognitive training approach***					
**Brockmeyer et al. 2019** [37]	*N* = 50 with BN or BED	Real ABM to avoid food cues vs. Sham ABM	10 sessions within 4 weeks	1. EDE (binge eating episodes)	1. Both groups had significantly fewer binge eating episodes after the training.
				2. FCQ (trait food craving, food cue reactivity)	2. Both groups reported significantly lower trait food craving and reduced food cue reactivity after the training.
				3. Bogus Taste Test (food intake)	3. There was no significant change in food intake in any group.
				4. AAT (approach and attention bias towards food)	4. There was no significant change in approach and attention bias towards food in any group.
**Giel et al. (2017)** [38]	*N* = 22 women with BED	Food specific inhibition training based on antisaccade paradigm vs. Control group with free vision instruction	3 individual sessions within 2 weeks	1. EDEQ (number of binge eating episodes in the last 4 weeks)	1. There were significantly lower numbers of binge eating episodes in both groups.
				2. FCQ (state version)	2. Reduced error rates and increase in food related inhibitory control in both groups.
				3. YFAS (food addiction total score)	3. No effect on food craving or food addiction were found in any group.
**Turton et al. (2018)** [39]	*n* = 27 women with BN and *n* = 17 with BED vs. lean and overweight controls	Food specific Go/No-Go training vs. General Go/No-Go training (within-subject-design)	1 individual session	1. Taste test for food consumption following the training	1. Small non-significant reductions in high-calorie food consumption in the food specific vs. the general training.
				2. 24- h post food diary including a sense of ‘loss of control’ and purging episodes	2. No treatment effect on binge eating or purging symptoms in the 24-h post diary.
				3. FCQ (food craving)	3. No treatment effect on food craving.
***Direct neuromodulation approach (neurostimulation and neurofeedback)***					
**Burgess et al. (2016)** [40]	*N* = 30 adults with BED or sub-BED	Real tDCS on DLPFC (anode right, cathode left) vs. sham tDCS on DLPFC (within-subject-design)	2 individual sessions	1.FPCT (Food craving) 2. In-lab food intake test	1. & 2. Food craving and food intake were reduced after tDCS compared to sham stimulation.
				3. 5-day at-home binge eating survey (urge to binge eat and binge eating frequency 5 days)	3. Urge to binge eat in men was reduced after tDCS vs. sham; no reduction concerning binge eating frequency in both conditions.
**Gay et al. (2016)** [41]	*N* = 47 women with BN	High frequency rTMS on left DLPFC vs. sham rTMS on left DLPFC	10 individual sessions over 2 consecutive weeks	1. Number of binge episodes in the last 15 days after stimulation	1. No significant reduction was found in any groups, and there was no difference between groups.
				2. Number of vomiting episodes in the last 15 days after stimulation	2. No significant reduction was found in any groups, and there was no difference between groups.
**Kekic et al. (2017)** [42]	*N* = 39 adults with BN	tDCS on DLPFC (anode right/cathode left) vs. tDCS on DLPFC (anode left/cathode right) vs. sham tDCS on DLPFC (within-subject-design)	2 individual sessions	1. Urge to binge eat on visual analogue scale	1. Both active conditions vs. sham show significant reduction in urge to bingeeat.
				2. FCT (food craving)	2. There was no group difference for food craving.
				3. Temporal Discounting (general reward processing)	3.Increased discounting in both active conditions vs. sham condition
				4. Self-reported binge eating and purging frequency 24 h after stimulation	4. No differences between conditions were found.
**Max et al. (2020)** [43]	*N =* 27 with BED	anodal 1 mA tDCS on DLPFC vs. sham tDCS (within-subject) vs. anodal2 mA tDCS on DLPFC vs. sham tDCS (within-subject design)	2 individual sessions	1. food-related antisaccade task (latency, error rate)	1. Significant reduction of error rate over time in all conditions; Latencies were decreased in the 2 mA vs. sham and vs. 1 mA condition.
				2. Frequency of binge eating episodes in the past seven days	2. Compared to sham stimulation, the frequency of binge eating episodes decreased at the 2 mA condition over time whereas it did not change significantly at the 1 mA condition.
**Schmidt & Martin (2016)** [44]	*N* = 75 healthy women with subjective binge eating episodes	EEG-neurofeedback with cue exposure vs. mental imagery with cue exposure vs. waitlist	10 individual sessions	1. EDEQ (binge eating episodes)	1. EEG-neurofeedback and MI groups showed decreased binge eating frequency, but this decrease was significant only at EEG-neurofeedback group at post test and 3-months follow up.
				2. FCQ (trait version)	2. Food craving was reduced in both EEG-neurofeedback (large effect) and MI groups (medium effect).
**Van den Eynde et al. (2010)** [45]	*N* = 38 adults with BN or EDNOS-bulimic type	High frequency rTMS on the left DLPFC vs. sham rTMS on the left DLPFC	1 individual session	1. Urge to eat, urge to binge eat, hunger on visual analogue scale immediately after stimulation	1. Urge to eat was significantly reduced in real rTMS group vs. sham stimulation. Urge to binge eat and hunger were reduced in both real rTMS and sham conditions.
				2. Binge eating frequency 24 h after stimulation	2. Significantly fewer binge-eating episodes over the 24 h following were reported in in real rTMS compared to sham.
				3. FCQ (state version)	3. Both groups reduced food craving, and there was no group difference.

*Note*. *AAT* Approach–Avoidance Task. *ABM* Approach Bias Modification, *BED* Binge Eating Disorder, *BES* Binge Eating Scale, *BIS/BAS* Behavioral Inhibition System/Behavioral Activation System Questionnaire, *BIS-15* Barrat Impulsiveness Scale-short version, *BN* Bulimia Nervosa; CBT, Cognitive Behavioural Therapy, *DEBQ* Dutch Eating Behaviour Questionnaire, *DLPFC* Dorsolateral Prefrontal Cortex, *EDE* Eating Disorders Examination Interview, *EDEQ* Eating Disorder Examination Questionnaire, *EDI* Eating Disorders Inventory, *EDNOS* Eating Disorder Not Otherwise Specified, *FCI* Food Craving Inventory, *FCQ* Food Craving Questionnaire, *FCT* Food Challenge Task, *FPCT* Food Photo Craving Test, *IBT* Intensive Behavioral Therapy, *MI* Mental Imagery, *OSFED* Other Specified Feeding or Eating Disorder, *rTMS* Repetitive Transcranial Magnetic Stimulation, *SST* Stop Signal Task, *sub-BED* sample with subthreshold BED, *TAU* Treatment as Usual, *tDCS* Transcranial Direct Current Stimulation, *TFEQ* Three-factor Eating Questionnaire, *YFAS* Yale Food Addiction Scale

## Results

**Fig. 1:**
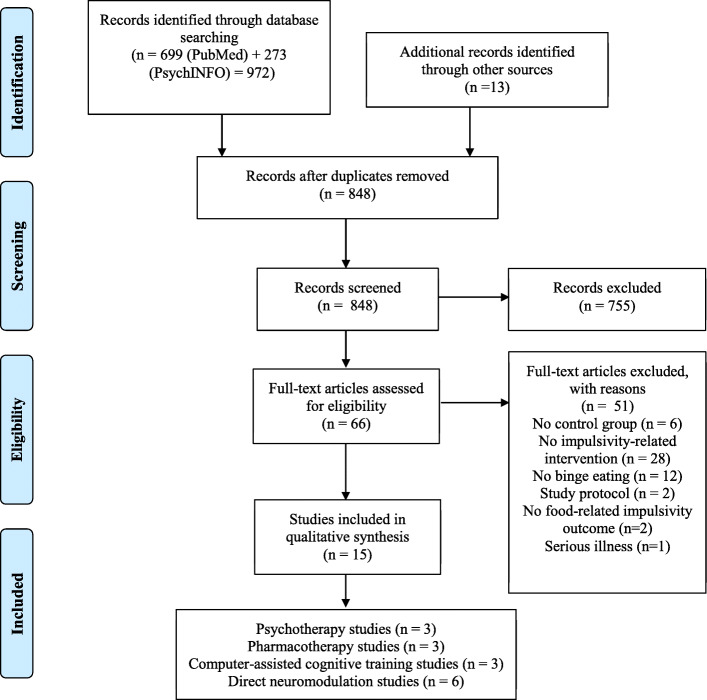
Flow diagram of study selection process for impulsivity-focused interventions to reduce binge eating behaviour

The detailed information regarding the selection procedure is presented in the flowchart in Fig. 1. 972 articles from the systematic search in PubMed and PsycINFO and 13 additional articles through hand search were identified. After the title/abstract screening, 66 studies remained for full-text investigation. Finally, 15 studies were analysed in this systematic review after excluding studies that did not fulfil one or more of the eligibility criteria. Participants were adults in all studies and included patients with an eating disorder diagnosis, with the exception of one study which included patients with subjective binge eating episodes [44]. Studies were categorised based on the treatment approach used: psychotherapy, pharmacotherapy, computer-assisted cognitive training, or direct neuromodulation interventions (i.e. neurostimulation and neurofeedback) (see Table 1).

### Interventions using a psychotherapy approach

Three studies were identified as including psychotherapy approaches [3, 32, 33]. One study investigated cue exposure therapy in virtual reality (VR-CET) as second-level treatment after CBT in patients with BN or BED, in comparison to additional cognitive behavioural therapy (A-CBT). The VR-CET was developed based on the classical conditioning model of binge eating to reduce food cravings by breaking the connection between the craved food(s) and binge eating behaviour. To achieve this, a virtual environment is simulated which depicts the usual location of binge eating episodes and includes exposures of their frequently consumed foods during binge eating episodes [3]. Even though participants in both treatment groups had improvements, participants in the VR-CET showed significantly greater abstinence rates from binge eating episodes and lower binge eating and purging frequency, as well as lower food craving, in comparison to the participants who received A-CBT. In the study by Preuss and colleagues [32], a novel treatment called ImpulsE, which strengthens emotion-based and food-related inhibitory control abilities, was compared with CBT as treatment as usual (TAU) for patients with obesity and a subgroup of patients with BED. The ImpulsE treatment included motivational techniques for change, emotion regulation skills and a food-specific, computer-assisted, Stop Signal inhibition training. Findings revealed a significant reduction in the frequency of overeating, perceived lack of perseverance and urgency in both conditions. However, significant binge eating reduction in patients with BED at post-treatment and 3-month follow up was found only in the ImpulsE group. The ImpulsE group also resulted in significantly greater reduction in inhibitory control compared with TAU, but no differences in trait-like impulsivity.

Schag and colleagues [33] investigated the efficacy of a newly developed cognitive-behavioural group treatment for impulsive eating in patients with BED called IMPULS. This treatment program mainly focusses on reducing impulsive eating behaviour and consists of several techniques such as food cue exposure, stimulus and response control. The IMPULS group was not superior to the control group receiving no intervention in terms of reducing binge eating episodes directly after treatment. However, the improvement in binge eating frequency at 3-month follow up continued only in the IMPULS group, reporting that binge eating frequency was more reduced compared to the control group at follow up. In terms of external eating, the IMPULS group showed improvement both at the end of treatment and follow up, whereas the control group showed an improvement only at follow up. Trait impulsivity was not reduced in either group. Taken together, these studies suggest that psychotherapy treatments that focus on impulsivity are fruitful in targeting food-related impulsive behaviour, but seem not to be able to change the impulsivity trait per se.

### Interventions using a pharmacotherapy approach

Three studies were identified investigating the efficacy of pharmacotherapy for binge eating [34–36]. The first study investigated whether liraglutide would be helpful to reduce reward sensitivity towards food and meal intake among people with obesity and binge eating behaviour [34]. Liraglutide is a glucagon-like peptide-1 (GLP-1) agonist reducing activation of brain areas associated with appetite and reward, and is used in the treatment of type 2 diabetes and obesity. For this purpose, participants were randomised to intensive behaviour therapy (IBT) alone, IBT-liraglutide, or multicomponent therapy including IBT, liraglutide, and portion-controlled diet. Binge eating episodes were significantly decreased in the IBT-liraglutide and multicomponent groups, and the multicomponent group was superior to the IBT-alone group at week 24. Although binge eating episodes were significantly decreased in all groups at week 52, the multicomponent group showed a greater reduction. In terms of food cravings and dietary disinhibition, all groups had significant within-group declines at both 24 and 52 weeks. However, the IBT-alone group showed significantly less decline in dietary disinhibition compared to the multicomponent group at week 24. Furthermore, a recent study also administered medications for the treatment of type 2 diabetes to treat binge eating behaviour [35]. The efficacy of dulaglutide, a GLP-1 receptor agonist that modulates appetite and reward-related brain areas, was compared to gliclazide, an anti-diabetic medication, in a sample of outpatients with type 2 diabetes and BED. Binge eating behaviour was only significantly reduced in in the dulaglutide group. In another study, Quilty and colleagues [36] tested the efficacy of methylphenidate, which is usually prescribed to treat ADHD, known to reduce impulsivity, and influence appetite and weight. They compared this medication with CBT as TAU. Findings revealed that both treatments, i.e. methylphenidate and CBT resulted in decreased binge eating. Furthermore, it was found that perseverance and negative urgency scores were decreased over time in both conditions. Higher levels of these scores at baseline assessment were associated with better treatment outcomes. The possible reasoning behind this finding is that the subjects with higher negative urgency and perseverance might have had experienced higher levels of stress. Thus, they might have had higher levels of treatment motivation and adherence. Since all these studies differed in methodology and participants had comorbidities, it is hard to comment on the efficacy of impulsivity-focused pharmacotherapy approaches. Findings of two studies comparing medication with psychotherapy [34, 36] did not provide evidence for a superiority of impulsivity-focused medication, although the coupling with psychotherapy seems promising and effective.

### Interventions using a computer-assisted cognitive training approach

There were three studies that included computer-assisted cognitive trainings for decreasing impulsivity symptoms among individuals with binge eating behaviour [37–39]. Two studies were based on decreased inhibitory control tendencies and therefore utilised food-related inhibitory control computer trainings for the treatment. Giel and colleagues [38] developed a food-specific inhibition training in an eye tracking antisaccade paradigm, i.e. looking at the opposite direction of the given stimulus as quickly as possible, for individuals with BED. The training was compared to a free vision condition in the control group. In their study, they found that both conditions resulted in a reduction of binge eating episodes and an increase in inhibitory control. However, the participants in the control condition looked at high-caloric food stimuli more often than the participants in the training group over all sessions. In another study, a food-specific and a non-food Go/no-go inhibition training with one session for each training condition was administered in a within-subjects design in women with BN or BED [39]. In the Go/no-go training, the patients are instructed to press a specific button when a go cue is shown and to withhold this response when a no-go cue is shown. In this study, neither the food-specific nor the general inhibition training led to a statistically significant decrease in food intake and both conditions did not differ concerning binge eating and purging symptoms. A more recent study [37] examined the efficacy of an approach bias modification (ABM) in which patients with BN or BED are trained to avoid visual cues of high-calorie foods. Participants receiving real ABM did not show significantly greater reductions in the number of objective binge eating episodes, trait food craving, and food cue reactivity than participants receiving sham ABM who were not trained to avoid food cues. Furthermore, no change in food intake, approach bias, and attention bias toward food was found in any group. Based on the available studies, there is hardly evidence that such computer-assisted cognitive trainings are effective for decreasing binge eating behaviour. This might be due to the low dose [39], due to the chosen paradigm in the control group which might also be effective [38], or due to not targeting the necessary underlying mechanisms of action [37].

### Interventions using a direct neuromodulation approach

Six studies were identified as directly targeting the brain for the treatment of impulsivity symptoms in people with binge eating behaviour. More specifically, one study used neurofeedback [44] whereas five studies investigated the effectiveness of non-invasive brain stimulation methods comparing verum with sham stimulation [40–43, 45].

Schmidt and Martin [44] tested the efficacy of EEG-neurofeedback combined with cue exposure against two comparison groups including mental imagery with cue exposure and a wait-list control group in healthy women with subjective binge eating episodes. They found that binge eating frequency decreased in both intervention groups. However, this decrease was only significant in the EEG-neurofeedback group. Furthermore, food craving was significantly reduced in the EEG-neurofeedback group (with a large effect) and the mental imagery with cue exposure group (with a medium effect) compared to the control group.

The neurostimulation studies targeted the dorsolateral prefrontal cortex (DLPFC) given its relationship with impulsivity and cognitive control. Among these neurostimulation studies, two tested the efficacy of repetitive transcranial magnetic stimulation (rTMS) [41, 45]. Van den Eynde and colleagues [45] compared verum rTMS (10 Hz) over the left DLPFC to sham in patients with bulimic disorders and reported less urge to eat immediately after stimulation and fewer binge eating episodes 24h after stimulation in the verum condition. However, Gay and colleagues [41] did not find a reduction after verum or sham rTMS (10 Hz) over the left DLPFC even after 10 stimulation sessions in terms of binging and purging episodes.

Furthermore, there were three studies administering transcranial direct current stimulation (tDCS) [40, 42, 43]. Burgess and colleagues [40] showed decreased in-lab food intake and food craving in patients with BED, and decreased urge to binge eat, particularly in men, following anodal verum tDCS administration (2 mA) over the right DLPFC in comparison to sham stimulation. However, no effect on binge eating frequency was found in any condition. Similarly, in the study of Kekic and colleagues [42], patients with BN received two stimulation conditions (anode right DLPFC/cathode left DLPFC; 2 mA, and anode left DLPFC/cathode right DLPFC; 2 mA) compared with sham condition in a counterbalanced order. In the two verum conditions, a suppressed urge to binge eat and increased self-regulatory control were demonstrated. A recent randomised proof-of-concept-study investigated the efficacy of anodal tDCS over the right DLPFC (1 mA vs. 2 mA vs. sham) combined with a food-modified antisaccade task to increase response inhibition skills in BED patients [43]. In the food-modified antisaccade task, participants were instructed to look as fast as possible on the opposite side of the screen when they saw a food picture. Significant improvement concerning the latency in the antisaccade task and reduced binge eating frequency was reported only for the 2 mA condition. The authors also report a learning effect concerning error rate over time in all three conditions suggesting that patients with BED can benefit from the repeated execution of a computer-assisted cognitive task addressing the underlying cognitive impairments, particularly response inhibition. To summarise, findings regarding the efficacy of rTMS concerning a reduction of binge eating episodes are mixed. However, there is slightly more consistent evidence that tDCS and EEG-neurofeedback combined with cue exposure appear to decrease binge eating and food craving.

## Discussion

The aim of this systematic review was to investigate the available interventions addressing impulsivity, particularly concerning impulsive eating behaviour among patients with binge eating behaviour. Following the database search, 15 studies were investigated under four categories based on the delivered treatment approaches: psychotherapy, pharmacotherapy, computer-assisted cognitive training, and direct neuromodulation interventions (i.e. neurostimulation and neurofeedback).

### Summary and interpretation of findings

Trials concerning impulsivity-focused psychotherapeutic treatments are surprisingly really scarce. The treatments included in this review appeared to decrease binge eating and impulsive eating behaviour among individuals who suffer from BED and BN significantly more in comparison to psychotherapeutic approaches without a specific focus on food-related impulsivity [3, 32]. However, studies did not add evidence for the efficacy of these treatment approaches on improvements of impulsivity as a trait.

The studies investigating the efficacy of pharmacotherapy to decrease binge eating episodes have different methodologies making it hard to draw a conclusion. Though Glucagon-like peptide-1 [34, 35] and methylphenidate [36] seem helpful to reduce food cravings, administration of these medications appears not to result in a greater improvement when compared to psychotherapeutic approaches [34, 36]. Moreover, review studies on pharmacological approaches for the treatment of binge eating suggest that topiramate as an antagonist of kainate/AMPA glutamate receptor is able to reduce binge eating frequency by suppressing appetite [46, 47]. Further, Lisdexamfetamine dimesylate (LDX) has been suggested to regulate the dopamine and noradrenaline neurotransmitter systems that are involved in eating behaviour and reward regulation, and thus decrease binge eating [48]. LDX is also the only approved medication for adults with BED by the US Food and Drug Administration (FDA) [49, 50]. Thus, both these drugs can also be considered to address food-related impulsivity, even though the impulsivity concept was not explicitly included in the articles as a supposed working mechanism.

There were three studies that used a computer-assisted cognitive training approach. Although studies administering a food-specific inhibition training to address food-related impulsivity among patients with binge eating seemed to increase inhibitory control, no evidence was found for the superiority of these approaches in decreasing binge eating episodes or food craving in comparison with control conditions [37–39]. It might be that the presentation of food stimuli itself – independent from the used training task - induces habituation effects likely regarded as one underlying mechanism for food cue exposure interventions (see below).

Most of the food-related impulsivity treatment studies identified in the current review were direct neuromodulation interventions. However, it is important to state here that neither the methodologies of the studies nor the findings were identical. The findings of the two studies with rTMS were mixed, showing no significant or superior effect in one study [41], but a significant effect in the 24 h following the treatment for the decrease in binge eating episodes in the other [45]. This makes it hard to draw any conclusions about the efficacy of rTMS. On the other hand, tDCS as another non-invasive neuromodulation approach seemed to be a more fruitful approach to decrease binge eating and food craving because the related studies delivered more consistent findings (significantly greater reduction in real conditions compared to sham conditions) [40, 42, 43]. However, it is important to note that all neuromodulation studies did not use an active intervention in the control condition, but only a sham stimulation which lowers the impact of these results. In contrast, when compared to an active control condition, a combination of EEG-neurofeedback and cue exposure [44] resulted in decreased binge eating frequency with significantly greater outcomes compared to control. The inclusion of an active control condition can be considered as particular strength for this study.

Regarding our first research question, it is plausible to conclude that impulsive eating behaviour might be changed through impulsivity-related interventions in individuals with binge eating episodes. Available approaches appear to be especially promising for decreasing binge eating symptoms. Although there is less evidence, there are supportive findings for improvements in food craving and inhibitory control following food-related impulsivity treatments. One major issue concerning the first research question is the lack of active interventions in the control conditions. Only six out of the 15 studies included in this systematic review had an active treatment group as a comparator. Nevertheless, in these six studies with active control conditions, the results suggest that impulsivity-focused treatment is at least not inferior to treatment as usual.

Moreover, it is difficult to make a comment about our second research question regarding whether one impulsivity-focused intervention is superior to the others to decrease binge eating episodes for several reasons. First, the detailed systematic search in two scientific databases revealed only 15 studies meeting the eligibility criteria. Second, although separated into four categories, the studies were heterogeneous regarding the treatment paradigms, the intensity of treatments (e.g, session numbers, delivery format etc.), and research methodology. Furthermore, none of the treatment approaches in these studies resulted in improvements in all the features of food-related impulsivity (e.g., binge eating episodes, impulsivity trait, food craving etc.).

Besides not having enough evidence to draw a distinct conclusion, food-related cue exposure can be considered as a promising approach as some studies that used this intervention technique, either in psychotherapy [33], virtual reality or neurofeedback [3, 44] found improvement in binge eating behaviour. The proposed mechanisms of cue exposure concerning impulsivity are multifaceted: cue reactivity and conditioning (e.g. [51]), habituation processes, increased self-efficacy, and self-control (e.g. [52]). Contextual cues related to food and eating might also lead appetitive conditioned responses [53], thus integrating virtual reality tools into cue exposure focused treatment modalities for binge eating might also be beneficial. However, further research is necessary to answer this question. Furthermore, investigating the efficacy of impulsivity-focusing medications would be valuable given their supposed mechanisms of suppressing appetite and regulating the reward pathway. Another fruitful approach that is worthy to be mentioned are neuromodulation techniques, in particular tDCS [40, 42, 43]. Moreover, combinations of several interventions seem to be very promising, either the combination of psychotherapy with computer-assisted cognitive trainings [32], pharmacotherapy with behavioural interventions [34], or neuromodulation with computer-assisted cognitive trainings [43]. For example, such neuromodulation interventions combined with well-elaborated, food-related computer training paradigms might target binge eating behaviour more directly and thus, enhance its effectivity (e.g. [54]).

Overall, it is quite surprising that such few studies have been published concerning this topic, especially such few psychotherapy studies. This might be due to the fact that psychotherapeutic trials are very time-consuming and complex. Studies concerning interventions specifically focusing on negative urgency are also lacking. It could be that some earlier conducted studies were labelled differently, for example negative urgency is a fairly new accepted impulsivity factor and studies might have been done before under the umbrella of emotion regulation strategies.

Concerning the addressed mechanisms, it could be that the efficacy of the intervention depends on the specific maintaining factor for the binge eating behaviour that is targeted (i.e., reward sensitivity, disinhibition or negative urgency). A patient engaging in binge eating behaviour which is mainly due to having high levels of reward sensitivity might not benefit from a treatment if it only focuses on inhibitory control. Moreover, people with subclinical binge eating, BED or BN might benefit differently from the impulsivity interventions, as demonstrated with the identified studies using rTMS for patients with BN or EDNOS-bulimic type. Thus, rTMS might provide a better treatment outcome in individuals who do not engage in purging behaviours, like in patients with BED. Otherwise, impulsivity-focused treatments could be helpful in specific patient subgroups, for example if first-line CBT fails [3] or in patients with increased trait impulsivity or comorbid mental disorders like addictive behaviours or ADHD that are related to impulsivity.

Taking together, it is reasonable to say that the findings for treating binge eating behaviour with a focus on food-related impulsivity are promising. However, it is important to bear in mind that research in this field is still in its infancy. The reported evidence is really heterogeneous and scarce, and publication bias cannot be ruled out, although some studies report negative findings that do not favour impulsivity-focused treatments. Thus, there are still many more studies needed to draw a concise conclusion concerning our research questions.

### Considerations for future research and clinical work

Findings of the present systematic review reveal that studies targeting food-related impulsivity are heterogeneous in many aspects. In this regard, future research may benefit from combining successful aspects of the available interventions as well as including active treatment control conditions rather than wait-list or sham control conditions only (see above).

Food-related impulsivity among patients with binge eating behaviour is suggested to have multiple components rather than being a single construct. More specifically, evidence underlines reward sensitivity, inhibitory control, and attentional bias towards food-related cues that can account for food-related impulsivity features [12]. Given that the studies included in this review focused on different aspects of impulsivity and provided mixed findings, it is possible to argue that identifying the underlying impulsivity aspect in each individual and then providing a more targeted intervention might increase treatment success. Another option would be to develop an impulsivity-focused treatment program that addresses all main components of impulsivity as these components often interact with each other. For example, high reward sensitivity towards food stimuli in combination with low inhibitory control skills may trigger a binge eating episode especially when also combined with negative mood in terms of negative urgency. Thus, an impulsivity-focused treatment might be most beneficial if it addresses these interrelations as well.

Moreover, future research is needed to compare impulsivity focused treatments with other active treatments, in particular concerning neurostimulation studies (see above). Last, it is of high importance to choose an adequate control condition [55] and this might also explain the negative findings in the computer-assisted cognitive training programmes.

## Conclusion

Even though first-line treatment modalities (e.g., CBT, IPT, and DBT) are available for binge eating behaviour, recovery is only achieved by 50% of treated patients as the treatments do not generally address important underlying and maintaining mechanism of binge eating behaviour, i.e. food-related impulsivity. With this systematic review, available novel approaches based on food-related impulsivity for binge eating behaviour were presented. In conclusion, although more research is needed, these interventions appear to be fruitful for future research and clinical attempts for the treatment of binge eating behaviour and related impulsive factors.

## Data Availability

Available from the corresponding author on request

## Abbreviations

ABM: Approach Bias Modification
ADHD: Attention Deficit Hyperactivity Disorder
BED: Binge Eating Disorder
BN: Bulimia Nervosa
CBT: Cognitive-Behavioral Therapy
DBT: Dialectical Behaviour Therapy
DLPFC: Dorsolateral Prefrontal Cortex
ED: Eating Disorder
EDE: Eating Disorders Examination
EDEQ: Eating Disorders Examination Questionnaire
EDNOS: Eating Disorder Not Otherwise Specified
EEG: Electroencephalography
GLP-1: Glucagon-like peptide-1
IBT: Intensive Behaviour Therapy
IPT: Interpersonal Therapy
LDX: Lisdexamfetamine Dimesylate
OFSED: Other Specified Feeding or Eating Disorders
RCT: Randomised Controlled Trial
rTMS: Repetitive Transcranial Magnetic Stimulation
TAU: Treatment as Usual
tDCS: Transcranial Direct Current Stimulation
VR-CET: Cue Exposure Therapy in Virtual Reality
